# A population-based propensity score matching analysis of risk factors and the impact on survival associated with refusal of cancer-directed surgery in patients with prostate cancer

**DOI:** 10.1038/s41598-024-60180-w

**Published:** 2024-04-25

**Authors:** Yuanyuan Tang, Yunliang Gao, Ruochen Zhang, Tao Li, Yaojing Yang, Li Huang, Yongbao Wei

**Affiliations:** 1 Department of Oncology, The Second Xiangya Hospital, Central South University, Changsha, China; 2 Key Laboratory of Diabetes Immunology (Central South University), Ministry of Education, National Clinical Research Center for Metabolic Disease, Changsha, China; 3 Department of Urology, The Second Xiangya Hospital, Central South University, No139. Renmin Road, Changsha, China; 4 Shengli Clinical Medical College of Fujian Medical University, Fuzhou, China; 5 Department of Urology, Fujian Provincial Hospital, Fuzhou, China; 6 Clinical Nursing Teaching and Research Section, The Second Xiangya Hospital, Central South University, Changsha, China

**Keywords:** Cancer-directed surgery, Cancer mortality, Propensity score matching, Prostate cancer, Seer, Survival, Cancer, Urology

## Abstract

Cancer-directed surgeries (CDS) play a crucial role in prostate cancer (PCa) management along with possible survival and therapeutic benefits. However, barriers such as socioeconomic factors may affect patients’ decision of refusing recommended CDS. This study aimed to uncover risk factors and the impact on survival associated with CDS refusal. We retrospectively reviewed the Surveillance, Epidemiology, and End Results database for patients diagnosed with PCa between 2000 and 2019. Multiple sociodemographic and clinical characteristics were extracted to assess predictors for physicians’ surgical recommendations and patients’ surgical refusal, respectively. Propensity score matching was performed to balance the covariates. The impact of surgical refusal on mortality risk was also investigated. A total of 185,540 patients were included. The physician’s recommendation of CDS was significantly influenced by the patient’s age, race, income, home location, diagnosis year, Gleason score, prostate-specific antigen (PSA), and TNM stage. About 5.6% PCa patients refused CDS, most of whom were older, non-White race, lack of partners, living outside of metropolitan areas, with higher PSA or lower clinical TNM stage. Patients who refused CDS had an increased risk of cancer-specific mortality and overall mortality than those who performed CDS. Physicians may weigh a host of sociodemographic and clinical factors prior to making a CDS recommendation. Patients’ refusal of recommended CDS affected survival and was potentially modifiable by certain sociodemographic factors. Physicians should fully consider the hindrances behind patients’ CDS refusal to improve patient-doctor shared decision-making, guide patients toward the best alternative and achieve better outcomes.

## Introduction

Prostate cancer (PCa) is the most commonly diagnosed cancer among men in the United States, with an estimated 3.5 million incident cases in 2022^1^. It is still the second leading cause of cancer-related deaths in males, just behind lung cancer^1^. The vast majority (85%) of PCa survivors are 65 years or older, only and < 1% are younger than 50^2^. Management algorithms vary based on the stage and grade of PCa as well as patients’ characteristics such as age, comorbidity, and personal preferences. Cancer-directed surgeries (CDS) play a crucial role in PCa management along with possible survival and therapeutic benefits^3,4^. For example, radical prostatectomy is regarded as a curative treatment for localized PCa or one part of multimodal therapy for advanced one. However, despite of well-defined treatment guidelines, a considerable number of patients are not recommended to perform CDS and the surgical outcomes remain inconsistent in those with CDS. Striking disparities in outcomes based on patient-level characteristics (e.g., demographic and socioeconomic variables) remain. For instance, PCa imposes a disproportionate burden on non-White patients as they experience more unfavorable tumor characteristics and a higher mortality (reaching up to three-fold risk) than their cohort counterparts ^2,5,6^. A significantly increased cancer-specific mortality (CSM) can also been noted in unmarried^7^, uninsured^8^ or rural^9^ PCa patients. Therefore, it is of great clinical significance to analyze the role and efficacy of CDS in treating PCa patients.

Respecting PCa patients’ choices is the highest priority, however, the choice of CDS might be also affected by the physician’s recommendation. Previous studies have demonstrated that primary care physician substantially influences the decision-making regarding PCa treatment and the type of treatment ^10,11^. Physician specialty type such as urologists or oncologists also affects the initiation of cancer-directed treatment^11^. Despite given clear benefits of recommended CDS, some PCa patients may still refuse these treatments due to various socioeconomic and demographic variables. As shown by prior analysis, age, race, marital status, insurance status, and income level are to be associated with surgery refusal^7,12–14^. Those without private insurance, or unmarried are less likely to undergo CDS. Surgery refusal could subsequently lead to an increased risk of overall mortality (OM) and CSM^7^. Particularly, given the less effect of sole CDS in late-stage PCa patients, the impact of refusing surgery may be underestimated in previous studies. Unfortunately, there is not enough evidence in the literature to suggest why these more vulnerable populations are more likely to refuse CDS as a cancer treatment.

The purpose of this study was to identify the demographic/socioeconomic variables associated with physician’s recommendations and patients’ refusal of CDS by using a large national cancer database. Additionally, we investigated the impact of CDS refusal on eventual survival. A better understanding of them will be of value to address the disparities in refusal and surgical outcomes as well.

## Methods

### Data source

This population-based cohort study was based on the national Surveillance, Epidemiology and End Results (SEER) database, which covers approximately 48.0% of the United States population (https://seer.cancer.gov/about/overview.html). Authorization was obtained from SEER to download PCa data for this study in June 2022. Within the SEER database (2000–2019), we identified and included all patients more than or equal to 18 years old with histologically confirmed PCa. Certain patients were excluded: cases with an unknown death certificate, autopsy only, or those who died before recommended surgery; a survival time of fewer than three months.

This study was exempt from local research ethics committee approval, considering that SEER data were de-identified and publicly available for research use.

### Study population and variables

Sociodemographic and clinical characteristics of each patient were extracted for analysis as in our previous study^15^. A brief description of these variables is presented as follows: age at diagnosis, year of diagnosis, race (black (African American), white (Caucasian), others (American Indian/AK Native, Asian/Pacific Islander)), marital status (married, single, and unknown), annual household income (< $65 000, ≥ $65 000, and unknown), residential location (large city, small city and missing value), Gleason score (≤ 6, = 7, and 8–10), serum prostate-specific antigen (PSA) value (≤ 0.10 ng/ml, ≥ 98.00 ng/ml, others), systemic therapy (yes, no, unknown) and longitudinal follow-up of vital status. The PCa stage was identified by the American Joint Committee on Cancer Tumor-Node-Metastasis (AJCC-TNM) stage, seventh edition.

To identify the variables affecting physicians’ decisions, we set up a case–control cohort between patients with recommended CDS and not. According to SEER Program Coding and Staging Manual (https://seer.cancer.gov/tools/codingmanuals/), CDS-recommended (CDSR) was defined as the following items: surgery performed, surgery unknown if performed or recommended but not performed due to unknown reason, and surgery recommended but not performed due to patient’s, patient’s family member’s or the patient’s guardian’s refusal. CDS-not recommended (CDSnR) represented those patients not recommended to undergo CDS by medical service providers, regardless of whether the patients underwent the surgery or not.

To determine the variables contributing to the patient’s refusal of recommended surgery, another grouped comparison was conducted between those who underwent CDS (CDS accepted = CDSA) or not (CDS not accepted = CDSnA). CDSA was defined as patient accepted surgery treatment (surgery performed). Moreover, cancer-specific mortality (CSM) and overall mortality (OM) were collected to evaluate the benefit of CDSA for PCa patients. “SEER cause-specific death classification” and “vital status recode” in the database were used to calculate CSM and OM, respectively.

### Statistics analysis

The statistical analyses consisted of three steps. Firstly, nonparametric independent-sample tests were used to compare two cohort groups (CDSR vs CDSnR, CDSA vs CDSnA) before and after propensity score matching (PSM). PSM was performed to adjust differences in potential covariates by a 1:1 matching ratio. A subset of variables was chosen for PSM matching: age, diagnosis year, race, marital status, income, and home location. PSA, GS, and AJCC stages were not adopted for matching due to > 50% missing records. Secondly, binary logistic regression in univariate and multivariable analyses were applied to determine the variables associated with CDS recommendation or CDS refusal, respectively. Thirdly, the Kaplan–Meier method and multivariable Cox proportional hazard models were used to analyze the impact of refusal of recommended CDS on CSM and OM. Adjusted model 1 adjusts for age, chemotherapy, radiotherapy, and systemic therapy. Adjusted model 2 adjusts for age, chemotherapy, radiotherapy, systemic therapy, race, partner, home, and income. Data analyses were performed by using SPSS version 27.0 (IBM, Armonk, NY, USA) and R software (R software for statistical computing, Vienna, Austria). A *p*-value < 0.05 was considered to be statistically significant.

**Figure 1:**
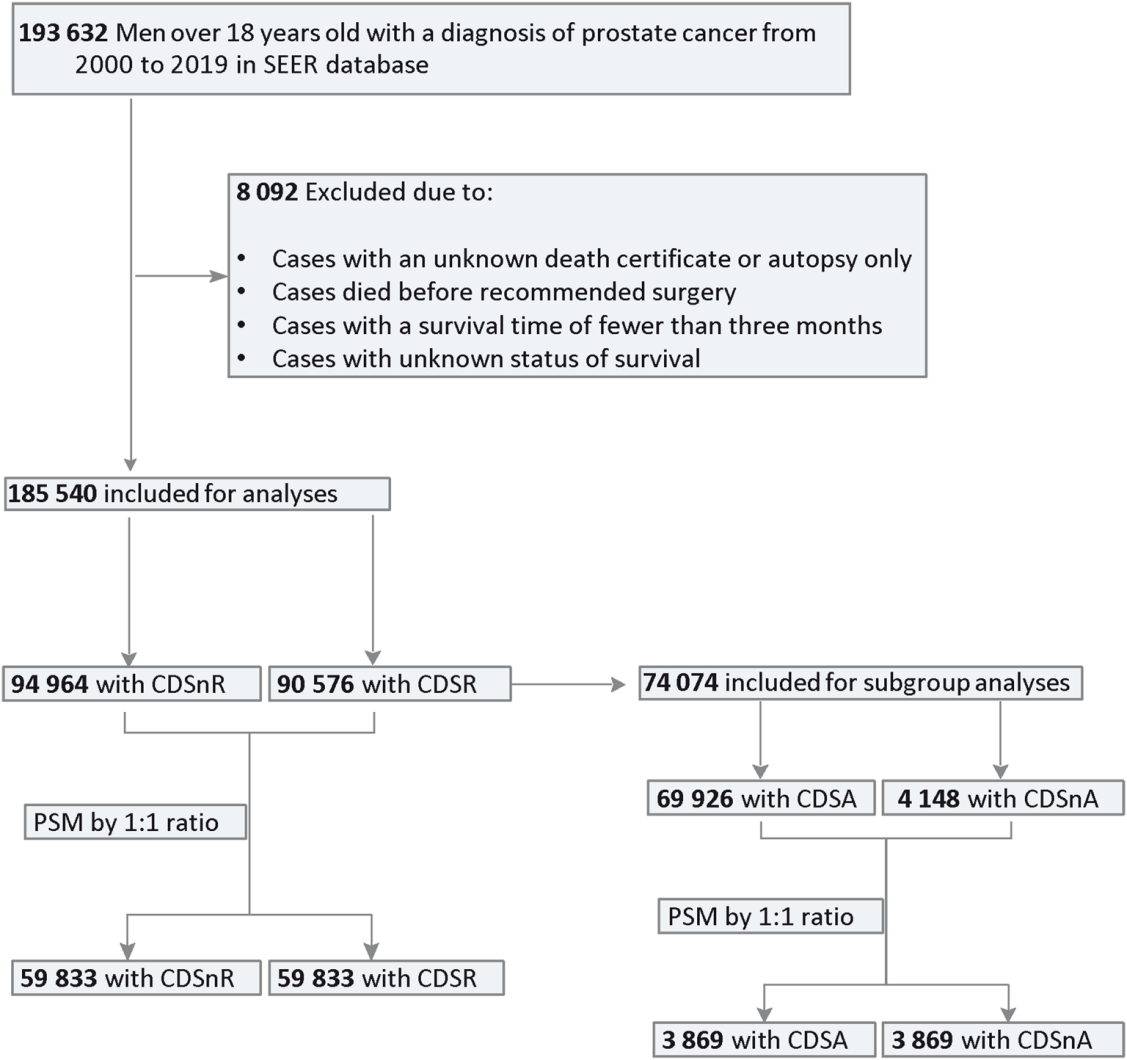
Study flowchart. Abbreviations: CDSR = cancer-directed surgery recommended; CDSnR = cancer-directed surgery not recommended; CDSA = cancer-directed surgery accepted; CDSnA = cancer-directed surgery not accepted; PSM = propensity score matching; SEER = the Surveillance, Epidemiology, and End Results.

A flow diagram (Fig. 1) shows the details of inclusion and exclusion criteria and the case–control study design.

### Ethical approval

This study was exempt from local research ethics committee approval, considering that SEER data were de-identified and publicly available for research use.

## Results

### Demographic and clinical characteristics of patients with or without CDS recommendation

A total of 193,632 PCa cases diagnosed between 2000 and 2019 were extracted from the database. After considering inclusion and exclusion criteria, 185,540 cases were finally included for analysis. The median age at diagnosis for the total study population was 60 to 69 years old (40.2%). Of these patients, 94,964 cases (51.2%) were recommended CDS and 90,576 (48.8%) were determined not to be surgical candidates (Table 1). The comparison without PSM showed significant differences in multiple covariates (age, race, partner, income, home location, diagnosis year, Gleason score, PSA, clinical M stage, chemotherapy, radiotherapy, and system therapy) between CDSR and CDSnR groups (all *p < *0.05). After PSM by 1:1 ratio, 59,833 cases were selected for each group. Significant differences could still be found in Gleason score, PSA, clinical TNM stage, chemotherapy, radiotherapy and system therapy between the two groups (all *p < *0.05).

**Table 1:** Baseline comparisons between the patients with cancer-directed surgery recommended (CDSR) and not recommended (CDSnR) from the Surveillance, Epidemiology, and End Results (SEER) database.

	Before PSM					After PSM				
	CDSR (N = 94,964)		CDSnR (90,576)		P	CDSR (n = 59,833)		CDSnR (n = 59,833)		P
	N	%	N	%		N	%	N	%	
Age (years)					.000					.753
30–39	78	.1	31	.0		18	.0	18	.0	
40–49	3640	3.8	1400	1.5		1105	1.8	1101	1.8	
50–59	24,480	25.8	13,699	15.1		10,723	17.9	10,734	17.9	
60–69	38,137	40.2	32,272	35.6		23,934	40.0	23,961	40.0	
70–79	23,008	24.2	34,419	38.0		19,573	32.7	19,617	32.8	
80 +	5621	5.9	8755	9.7		4480	7.5	4402	7.4	
Race					.000					.574
White	80,998	85.3	69,781	77.0		49,594	82.9	49,520	82.8	
Black	7694	8.1	12,064	13.3		6276	10.5	6323	10.6	
Other	5398	5.7	7363	8.1		3963	6.6	3990	6.7	
Unknown	874	.9	1368	1.5		–	–	–	–	
Partner					.000					.771
Married	68,512	72.1	56,802	62.7		45,556	76.1	45,513	76.1	
Single	17,263	18.2	20,090	22.2		14,277	23.9	14,320	23.9	
Unknown	9189	9.7	13,684	15.1		–	–	–	–	
Income (UDS)					.000					.804
0–$74,999	55,335	58.3	42,953	47.4		31,109	52.0	31,152	52.1	
$75,000 +	39,509	41.6	47,592	52.5		28,724	48.0	28,681	47.9	
Unknown	120	.1	31	.0		–	–	–	–	
Home					.000					.804
Metropolitan areas	38,010	40.0	54,880	60.6		30,304	50.6	30,347	50.7	
Others	56,834	59.8	35,665	39.4		29,529	49.4	29,486	49.3	
Unknown	120	.1	31	.0		–	–	–	–	
Diagnosis year					.000					0.702
2000–2009	84,764	89.3	73,875	81.6		52,622	87.9	52,665	88.0	
2010–2019	10,200	10.7	16,701	18.4		7211	12.1	7168	12.0	
Gleason score					.000					.000
≤ 6	3375	3.6	4487	5.0		2295	3.8	1413	2.4	
7	2670	2.8	2961	3.3		1853	3.1	1077	1.8	
8–10	1565	1.6	3953	4.4		1123	1.9	2152	3.6	
Missing	87,354	92.0	79,175	87.4		54,562	91.2	55,191	92.2	
PSA (ng/ml)					.000					.000
≤ 0.1	68	.1	27	.0		53	.1	17	.0	
≥ 98.0	756	.8	5290	5.8		631	1.1	3345	5.6	
Results not in chart	1711	1.8	3359	3.7		1198	2.0	1161	1.9	
Missing	92,429	97.3	81,900	90.4		57,951	96.9	55,310	92.4	
T stage					.105					.046
T1	608	.6	829	.9		447	.7	364	.6	
T2	1031	1.1	2031	2.2		679	1.1	652	1.1	
T3	243	.3	192	.2		173	.3	109	.2	
T4	43	.0	147	.2		31	.1	85	.1	
Missing	93,039	98.0	87,377	96.5		58,503	97.8	58,623	98.0	
N stage					.223					.039
Nx	320	.3	1049	1.2		147	.2	365	.6	
N0	1595	1.7	2598	2.9		1155	1.9	962	1.6	
N1	108	.1	575	.6		91	.2	372	.6	
Missing	92,941	97.9	86,354	95.3		58,440	97.7	58,134	97.2	
M stage					.000					.000
M0	1901	2.0	2936	3.2		1291	2.2	869	1.5	
M1	122	.1	1286	1.4		102	.2	830	1.4	
Missing	92,941	97.9	86,354	95.3		58,440	97.7	58,134	97.2	
Chemotherapy					.000					.000
Yes	564	.6	1624	1.8		374	.6	1153	1.9	
No	94,400	99.4	88,952	98.2		59,459	99.4	58,680	98.1	
Radiotherapy					.000					.000
Beam radiation	9423	9.9	26,699	29.5		6822	11.4	19,355	32.3	
Beam + implants/isotopes	1976	2.1	8567	9.5		1465	2.4	6493	10.9	
Radioactive implants	3276	3.4	15,020	16.6		2171	3.6	11,943	20.0	
Others	212	.2	713	.8		138	.2	507	.8	
Unknown	80,077	84.3	39,577	43.7		49,237	82.3	21,535	36.0	
Systemic therapy					.000					.000
Yes	2658	2.8	610	.7		1967	3.3	441	.7	
No	35,657	37.5	46,311	51.1		22,011	36.8	27,733	46.4	
Missing	56,649	59.7	43,655	48.2		35,855	59.9	31,659	52.9	
Cancer-specific mortality					.000					.000
Alive or dead of other cause	87,043	91.7	77,199	85.2		54,245	90.7	50,684	84.7	
Dead	7921	8.3	13,377	14.8		5588	9.3	9149	15.3	
Overall mortality					.000					.006
Alive	61,006	64.2	47,405	52.3		35,161	58.8	31,270	52.3	
Dead	33,958	35.8	43,171	47.7		24,672	41.2	28,563	47.7	
Survival months					.000					.000
Median (IQR)	143.00 (93.00–182.00)		122.00 (51.00–157.00)			138.00(80.00–179.00)		129.00 (65.00–164.00)		
Mean ± SD (Range)	134.56 ± 62.92 (1–239)		109.63 ± 63.57 (1–239)			129.13 ± 64.29 (1–239)		118.02 ± 63.06 (1–239)		

PSA, Prostate-specific antigen; PSM, propensity score matching.

Particularly, patients in the CDSR group presented lower rates in CSM (8.3% vs. 14.8%) and OM (35.8% vs. 47.7%), and longer median survival time (143.0 vs. 122.0 months), all of which remained similar after PSM (9.3% vs 15.3%, 41.2% vs 47.7%, 138.0 vs. 129.0 months, respectively) (all *p < *0.05).

### Factors predicting physician’s CDS recommendation

Univariate analysis (Table 2) demonstrated that the physician’s recommendation of CDS was influenced by the patient’s age, race, income, home location, diagnosis year, Gleason score, PSA and TNM stage. Patients being younger, White in the race, living with a partner, living in outside of metropolitan area, with lower income, lower PSA, lower TNM stage but with Gleason score = 7 would be preferentially recommended CDS treatments by physicians. On multivariate analysis, only age, Gleason score, and clinical T and M stages were significantly associated with the increased recommendation of CDS treatment.

**Table 2:** Factors related to physicians’ recommendation of cancer-specific surgery without propensity score matching.

	Univariate regression analysis						Multivariate regression analysis					
	B	Wald	p	Exp(B)	95% C.I.for EXP(B)		B	Wald	p	Exp(B)	95% C.I.for EXP(B)	
					Lower	Upper					Lower	Upper
Age (years)		7214.862	.000					12.174	.016			
30–39	−.033	.023	.879	.968	.635	1.474	1.072	5.829	.016	2.921	1.224	6.974
40–49	.342	2.591	.107	1.408	.928	2.136	1.057	6.172	.013	2.877	1.250	6.621
50–59	.756	12.654	.000	2.129	1.404	3.229	1.342	9.551	.002	3.825	1.634	8.957
60–69	1.325	38.912	.000	3.764	2.482	5.709	.753	2.371	.124	2.124	.814	5.540
70–79	1.366	41.117	.000	3.919	2.581	5.950	–	–	–	–	–	–
Race		1944.658	.000					.828	.661			
White	.599	1497.119	.000	1.820	1.766	1.876	.144	.573	.449	1.154	.796	1.674
Black	.460	607.175	.000	1.583	1.526	1.642	−.122	.177	.674	.885	.503	1.559
Partner		2068.502	.000									
Married	.339	821.881	.000	1.404	1.372	1.437	.204	1.540	.215	1.227	.888	1.694
Income (UDS)												
0–$74,999	.439	2202.475	.000	1.552	1.524	1.581	.159	.824	.364	1.173	.831	1.655
Home		7742.931	.000									
Metropolitan areas	−.833	7700.724	.000	.435	.427	.443	−.327	3.463	.063	.721	.511	1.018
Diagnosis year												
2000–2009	.631	2169.868	.000	1.879	1.830	1.929	–	–	–	–	–	–
Gleason score		459.410	.000					10.469	.005			
≤ 6	−.181	26.704	.000	.834	.779	.894	−.466	5.290	.021	.628	.422	.933
7	.642	291.902	.000	1.900	1.765	2.045	.128	.272	.602	1.136	.703	1.837
PSA (ng/ml)		756.426	.000					17.089	.000			
≤ 0.1	2.869	154.584	.000	17.623	11.211	27.702	2.878	12.693	.000	17.779	3.650	86.601
≥ 98.0	1.598	48.539	.000	4.944	3.154	7.751	2.242	8.006	.005	9.412	1.992	44.483
T stage		110.046	.000					47.659	.000			
T1	.368	31.389	.000	1.445	1.270	1.643	.120	.431	.512	1.128	.788	1.615
T2	−.546	24.452	.000	.579	.467	.719	-1.323	30.867	.000	.266	.167	.425
T3	.919	25.674	.000	2.507	1.757	3.578	−.882	6.987	.008	.414	.215	.796
N stage		188.633	.000						.366			
Nx	−.699	96.100	.000	.497	.432	.571	.333	2.010	.215	1.395	.825	2.360
N0	.485	15.600	.000	1.624	1.277	2.066	.437	1.541	.176	1.548	.822	2.916
M stage								1.829				
M0	1.921	374.833	.000	6.825	5.619	8.290	1.108	25.794	.000	3.028	1.975	4.644

PSA, Prostate-specific antigen.

### Demographic and clinical characteristics of patients with or without accepted CDS

Of the entire cohort, 74,074 cases were involved for comparison. The median age range was 60 to 69 years old (39.0%). 69,926 (94.4%) patients accepted CDS but 4148 (5.6%) refused CDS (Table 3). Before PSM, the comparison between CDSA and CDSnA showed significant differences in diverse covariates (age, race, partner, home location, diagnosis year, PSA, clinical M stage, radiotherapy and system therapy) (all *p < *0.05). After PSM, 3869 cases were left in each group for comparison. Significant differences remained in PSA, chemotherapy, radiotherapy and system therapy when comparing the two groups (all *p < *0.05).

**Table 3:** Baseline comparisons between the patients with cancer-directed surgery accepted (CDSA) and not accepted (CDSnA) from the Surveillance, Epidemiology, and End Results (SEER) database.

	Before PSM					After PSM				
	CDSA (N = 69,926)		CDSnA (4148)		P	CDSA (n = 3869)		CDSnA (n = 3869)		P
	N	%	N	%		N	%	N	%	
Age (years)					.000					1.000
30–39	72	.1	0	0		–	–	–	–	
40–49	3259	4.7	101	2.4		93	2.4	93	2.4	
50–59	21,106	30.2	761	18.3		710	18.4	710	18.4	
60–69	29,790	42.6	1617	39.0		1519	39.3	1519	39.3	
70–79	12,914	18.5	1449	34.9		1348	34.8	1348	34.8	
80 +	2785	4.0	220	5.3		199	5.1	199	5.1	
Race					.000					1.000
White	59,657	85.3	3340	80.5		3117	80.6	3117	80.6	
Black	5787	8.3	495	11.9		479	12.4	479	12.4	
Other	3978	5.7	298	7.2		273	7.1	273	7.1	
Unknown	504	.7	15	.4		–	–	–	–	
Partner					.000					1.000
Married	53,970	77.2	2818	67.9		2809	72.6	2809	72.6	
Single	12,484	17.9	1066	25.7		1060	27.4	1060	27.4	
Unknown	3472	5.0	264	6.4		–	–			
Income (UDS)					.092					1.000
0–$74,999	39,444	56.4	2394	57.7		2225	57.5	2225	57.5	
$75,000 +	30,447	43.5	1750	42.2		1644	42.5	1644	42.5	
Unknown	35	.1	4	.1		–	–			
Home					.000					1.000
Metropolitan areas	32,013	45.8	1595	38.5		1500	38.8	1500	38.8	
Others	37,878	54.2	2549	61.5		2369	61.2	2369	61.2	
Unknown	35	.1	4	.1		–	–			
Diagnosis year					.000					1.000
2000–2009	61,850	88.5	4029	97.1		3771	97.5	3771	97.5	
2010–2019	8076	11.5	119	2.9		98	2.5	98	2.5	
Gleason score					.076					0.254
≤ 6	2619	3.7	19	.5		32	.8	12	.3	
7	2162	3.1	28	.7		21	.5	21	.5	
8–10	1168	1.7	14	.3		19	.5	11	.3	
Missing	63,977	91.5	4087	98.5		3797	98.1	3825	98.9	
PSA (ng/ml)					.000					0.002
≤ 0.1	63	.1	2	.0		1	.0	2	.1	
≥ 98.0	519	.7	46	1.1		8	.2	42	1.1	
Results not in chart	1305	1.9	18	.4		14	.4	12	.3	
Missing	68,039	97.3	4082	98.4		3846	99.4	3813	98.6	
T stage					.584					0.934
T1	531	.8	4	.1		5	.1	3	.1	
T2	804	1.1	6	.1		8	.2	3	.1	
T3	240	.3	1	.0		1	.0	1	.0	
T4	42	.1	0	0		–	–	–	–	
Missing	68,309	97.7	4137	99.7		3855	99.6	3862	99.8	
N stage					.571					0.402
Nx	101	.1	7	.2		2	.1	4	.1	
N0	1462	2.1	9	.2		14	.4	6	.2	
N1	95	.1	6	.1		–	–	5	.1	
Missing	68,268	97.6	4126	99.5		3853	99.6	3854	99.6	
M stage					.000					0.061
M0	1563	2.2	16	.4		15	.4	10	.3	
M1	95	.1	6	.1		1	.0	5	.1	
Missing	68,268	97.6	4126	99.5		3853	99.6	3854	99.6	
Chemotherapy					.061					0.589
Yes	411	.6	15	.4		17	.4	14	.4	
No	69,515	99.4	4133	99.6		3852	99.6	3855	99.6	
Radiotherapy					.000					.000
Beam radiation	4159	5.9	1220	29.4		266	6.9	1168	30.2	
Beam + implants/isotopes	247	.4	556	13.4		23	.6	545	14.1	
Radioactive implants	309	.4	1022	24.6		32	.8	970	25.1	
Others	69	.1	22	.5		5	.1	19	.5	
Unknown	65,142	93.2	1328	32.0		3543	91.6	1167	30.2	
Systemic therapy					.000					.000
Yes	2637	3.8	1	.0		143	3.7	1	.0	
No	28,630	40.9	1213	29.2		1342	34.7	1101	28.5	
Missing	38,659	55.3	2934	70.7		2384	61.6	2767	71.5	
Cancer-specific mortality					.000					.000
Alive or dead of other cause	65,419	93.6	3645	87.9		3529	91.2	3408	88.1	
Dead	4507	6.4	503	12.1		340	8.8	461	11.9	
Overall mortality					.000					.000
Alive	50,041	71.6	2093	50.5		2272	58.7	1946	50.3	
Dead	19,885	28.4	2055	49.5		1597	41.3	1923	49.7	
Survival months					.038					.000
Median (IQR)	146.00 (107.00–184.00)		148.00 (101.00–190.00)			145.00 (1–6.00–181.00)		149.00 (102.00–192.00)		
Mean ± SD (Range)	138.75 ± 61.03 (1–239)		140.74 ± 62.09 (1–239)			137.74 ± 59.02 (1–239)		141.73 ± 62.18		

PSA, Prostate-specific antigen; PSM,  propensity score matching.

Of note, patients who refused CDS treatment had higher rates in CSM (12.1% vs. 6.4%) and OM (49.5% vs. 28.4%), but a slightly longer median survival time (148.0 vs. 146.0 months), all of which were quite similar after PSM (11.9% vs 8.8%, 49.7% vs. 41.3%, 149.0 vs. 145.0 months, respectively) (all *p < *0.05).

### Factors predicting patient’s refusal of CDS treatment

As shown by univariate analysis (Table 4), patients refusing CDS treatment were more likely to be older, non-White race, lack partners, living outside of metropolitan area, higher PSA, lower clinical N and M stage and were diagnosed before the 2009 year (all *p < *0.05). However, multivariate analysis indicated no factors significantly related to patients’ refusal of CDS treatment.

**Table 4:** Factors related to patients’ refusal of cancer-specific surgery without propensity score matching.

	Univariate regression analysis						Multivariate regression analysis					
	B	Wald	p	Exp(B)	95% C.I.for EXP(B)		B	Wald	p	Exp(B)	95% C.I.for EXP(B)	
					Lower	Upper					Lower	Upper
Age (years)		754.453	.000					.000	1.000			
30–39	17.758	.000	.997	51,540,522.402	.000		−.030	.000	1.000	.970	.000	
40–49	17.909	.000	.997	59,963,990.376	.000		2.082	.000	1.000	8.018	.000	
50–59	18.318	.000	.997	90,271,637.294	.000		-4.446	.000	.999	.000	.000	
60–69	19.044	.000	.997	186,603,337.136	.000		-4.067	.000	.999	.000	.000	
70–79	18.694	.000	.997	131,373,957.914	.000		–	–	–	–	–	–
Race		86.151	.000					.206	.902			
White	.424	71.590	.000	1.528	1.385	1.685	1.140	.206	.650	3.128	.023	430.768
Black	.291	21.614	.000	1.338	1.183	1.513	−.250	.000	1.000	.779	.000	
Partner												
Married	.492	173.855	.000	1.635	1.520	1.759	−.260	.010	.919	.771	.005	114.419
Income (UDS)												
0-$74,999	−.054	2.831	.092	.947	.889	1.009	1.223	.182	.669	3.399	.012	934.972
Home												
Metropolitan areas	.301	83.907	.000	1.351	1.267	1.440	-5.623	.000	.996	.000	.000	
Diagnosis year												
2000–2009	-1.486	251.286	.000	.226	.188	.272	–	–	–	–	–	–
Gleason score		4.031	.133					.000	1.000			
≤ 6	.580	3.766	.052	1.785	.994	3.205	15.108	.000	.996	3,643,630.658	.000	
7	.502	2.012	.156	1.652	.826	3.307	−.915	.000	1.000	.401	.000	
PSA (ng/ml)		.312	.000					.000	1.000			
≤ 0.1	1.027	.000	.162	2.792	.662	11.780	14.360	.000	.999	1,723,150.969	.000	
≥ 98.0	−.834	.279	.270	.434	.099	1.914	-3.357	.000	1.000	.035	.000	
T stage		.000	.958					.320	.956			
T1	−.009	.648	.988	.991	.278	3.527	16.265	.000	.997	11,584,247.088	.000	
T2	−.592	1.121	.597	.553	.061	4.975	14.865	.000	.997	2,855,437.738	.000	
T3	-16.314	6201.910	.998	.000	.000		-1.841	.000	1.000	.159	.000	
N stage		29.203	.000					.248	.883			
Nx	-2.421	22.158	.000	.089	.032	.243	15.887	.000	.999	7,934,962.122	.000	
N0	−.093	.026	.872	.911	.296	2.809	17.820	.000	.998	54,858,377.907	.000	
M stage												
M0	1.820	13.777	.000	6.170	2.360	16.127	15.416	.000	.997	4,954,843.115	.000	

PSA,  Prostate-specific antigen.

### Refusal of CDS and Mortality

**Figure 2:**
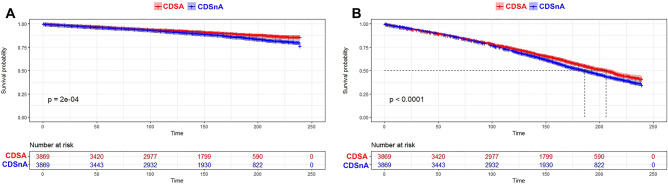
Impact of surgical refusal on survival rate in unselected prostate cancer patients from SEER data base between 2010 and 2019. Shown are. (**A**) Kaplan–Meier curves of cancer-specific survival in patients with prostate cancers. (**B**) Kaplan–Meier curves of overall survival in patients with prostate cancers. (All *p < *0.001) Abbreviations: CDSA = cancer-directed surgery accepted; CDSnA = cancer-directed surgery not accepted.

**Figure 3:**
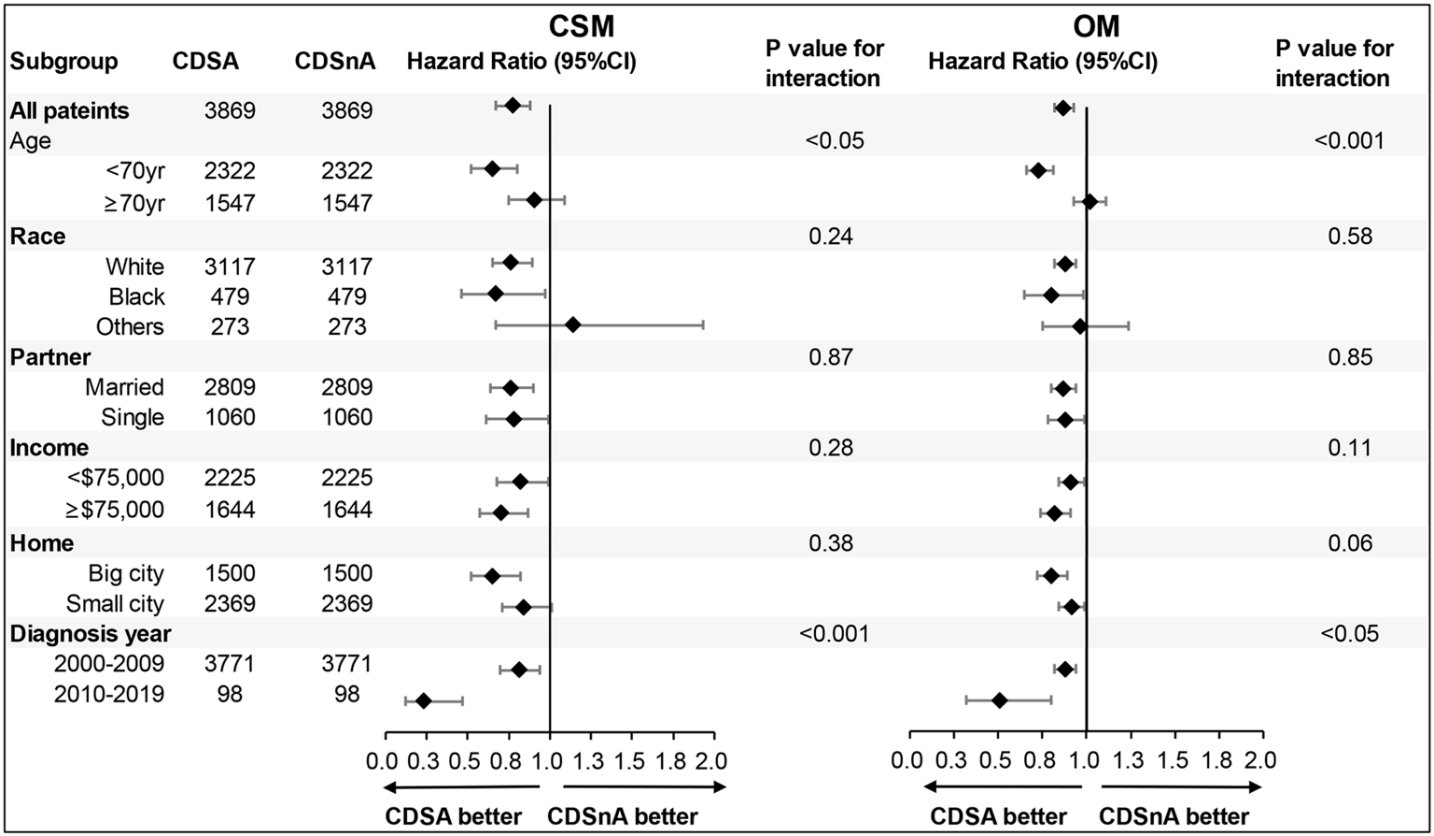
Forest plot the subgroup analysis for sociodemographic factors and prostate cancer in cancer-specific mortality (CSM) and overall mortality (OM), respectively. Abbreviations: CDSA = cancer-directed surgery accepted; CDSnA = cancer-directed surgery not accepted.

To further investigate the impact of the decision to refuse surgery on survival, Kaplan–Meier analysis and multivariable Cox proportional hazard models were adopted. As shown in Kaplan–Meier plots (Fig. 2A,B), significantly lower rates of CSM and OM (both *p < *0.05) were determined in the CDSA group after 120 months. Cox proportional hazard models with or without PSM supported that CDS refusal could significantly increase the risk of CSM (hazard ratio, 0.54; 95% confidence interval, 0.49–0.59) and OM (hazard ratio, 0.59; 95% confidence interval, 0.56–0.61), respectively (Table 5). The Forest plot presented the subgroup analysis for CDSA vs CDSnA in CSM and OM, respectively (Fig. 3). The results demonstrated that patients refusing CDS obtained significantly poorer prognoses than those accepting CDS, particularly across age and diagnosis year subgroups. Younger patients diagnosed between 2010 and 2019 were more likely to have lower rates of CSM and OM.

**Table 5:** Multivariable Cox proportional hazard model for CSM and OM for patients with CDSA based on patients with CDSnA.

Outcomes	CDSA HR (95% CI)	*P*-value
CSM		
Non-adjusted	0.54 (0.49–0.59)	*p < *0.001
Adjusted model 1	1.26 (0.87–1.82)	p = 0.23
Adjusted model 2	1.36 (0.93–1.98)	p = 0.12
PSM Non-adjusted	0.77 (0.67–0.88)	*p < *0.001
PSM Adjusted model 1	1.79 (0.87–3.69)	p = 0.12
PSM Adjusted model 2	2.18 (1.04–4.55)	*p < *0.05
OM		
Non-adjusted	0.59 (0.56–0.62)	*p < *0.001
Adjusted model 1	0.99 (0.81–1.21)	*p* = 0.93
Adjusted model 2	1.07 (0.87–1.32)	*p* = 0.52
PSM Non-adjusted	0.87 (0.82–0.93)	*p < *0.001
PSM Adjusted model 1	0.96 (0.59–1.55)	*p* = 0.87
PSM Adjusted model 2	1.01 (0.67–1.75)	*p* = 0.75

Non-adjusted model adjusts for none.

Adjusted model 1 adjusts for age, chemotherapy, radiotherapy, and systemic therapy.

Adjusted model 2 adjusts for age, chemotherapy, radiotherapy, systemic therapy, race, partner, home, and income.

Abbreviations: HR = hazard ratio; PSM = propensity score matching (by1:1 matching); CI = confidence interval; CSM = cancer-specific mortality; OM = overall mortality.

## Discussion

Our study presented one of the largest pooled analyses of patients with PCa and highlighted the identifiable factors to predict the likelihood of a physician’s CDS recommendation and a patient’s CDS refusal. Particularly, we demonstrated that CDS refusal was associated with increased odds of CSM and OM. A better understanding of the effects of sociodemographic factors may enable to improve patients’ satisfaction, surgical utilization and treatment outcomes.

The decision to undergo CDS in PCa patients is personal and complex and undoubtedly, patients have full rights in their decision-making process. However, our results revealed that physicians’ recommendation of CDS could strongly affect patients’ final choices. Univariate analysis in this study demonstrated that physician’s recommendation of CDS was determined by the patient’s age, race, income, home location, diagnosis year, Gleason score, PSA and TNM stage. However, after multivariate analysis, only age, Gleason score, and clinical T and M stages were significantly associated with the increased recommendation of CDS treatment. In other words, physicians may only factor patients’ medical situation into their CDS recommendation and this decision process was not affected by patients’ socioeconomic factors. This finding was compatible with previous literature. Scherr et al. reported that PCa patients’ treatment decisions were chiefly decided by their urologists’ recommendations, which in turn were driven by medical factors (age, Gleason score, etc.) without patients’ preferences^16^. In addition, PCa patients diagnosed by urologists, rather than radiation oncologists, greatly preferred to receive up-front treatment such as CDS^10^. Different specialty types could lead to disparities in treatment outcomes in PCa patients^11^. Given the centrality of physicians’ recommendations in the decision-making process, physicians should strive for effective communication with the candidates and emphasize the important role of CDS in managing PCa.

CDS, such as radical prostatectomy and cytotherapeutic ablation, could serve as an established pillar of therapeutic options for PCa in particular localized ones^3,4^. When facing the selection of surgical interventions, patients undeniably weigh the potential tradeoffs between benefits and burdens. Despite the potential lifesaving or life-prolonging effect of CDS, a portion of PCa patients may still refuse to receive CDS treatments due to multiple reasons. Our study reported that about 5.6% PCa patients refused CDS, most of whom were older, non-White race, lack of partners, living outside of metropolitan areas. Particularly, PCa patients with higher PSA or lower clinical TNM stage proposed CDS refusal. These results were in parallel with prior studies. Islam et al. found that about 3.9% PCa patients refused the suggested surgery and those black, single, Medicaid/Medicare-covered, or early-stage ones had significantly increased odds of refusal rate^12^. Xu and colleagues reported a relatively lower refusal rate (2.47%) of CDS in PCa patients and pointed out that black and Asian/Pacific Islander patients were more likely to refuse CDS than White ones^14^. Quiet similarly, a recent study by Dee et al. indicated that older age, black/Asian, noninsurance or Medicaid, community facility type and later year of diagnosis were associated with increased odds of locoregional treatment (i.e., surgery ) refusal in PCa patients^13^. The influence of sociodemographic factors on CDS refusal can also be found in other cancer treatments such as lung cancer^17^, colon cancer^18^, breast cancer^19^ and so on. More attention should be paid to these factors that influence patients’ treatment decisions.

Sociodemographic factors especially age, race, marital status and cancer stage could act as vital predictors for patients’ CDS refusal due to nuanced and complex contributions. For instance, older patients may be more likely to refuse CDS due to a fear of a decrease in quality of life^7^, a perceived lack of social support^20^, an unaffordable surgical fee^21^, a group of comorbidities^22^, an existing communication gap between physicians^23^ and so on. Besides, the high rate of CDS refusal in non-White populations might be attributed to greater distrust toward the healthcare system^24^, late lacking medical insurance^12^ and different cultural competency^25^. Consequently, sociodemographic factors can play a crucial role in the decision of declining the CDS for PCa patients. Physicians, especially urologists, should fully consider the barriers behind patients’ refusal of CDS to improve patients’ satisfaction, surgical utilization, and treatment outcomes.

Of note, the most influential factors in PCa patients’ treatment decisions were the perceptions of therapeutic efficacy and side effects, mainly derived from physicians’ descriptions^26^. Our study revealed that PCa patients who refused CDS had an increased risk of death ( hazard ratio 0.54 in CSM and hazard ratio 0.59 in OM) than those who accepted. Consistently, Rapp et al.’s study identified an overall 1.60 higher mortality in PCa patients who refused CDS^7^. In other words, PCa patients could significantly benefit from CDS and achieve a longer survival time. Given surgery refusal increasing CSM, physicians should carefully and clearly inform PCa patients regarding their prognosis in case they are thinking of skipping surgical treatment. CDS may be a viable alternative option for those with locally advanced or even distant stages of PCa.

Admittedly, certain limitations in this study should be addressed. Above all, the retrospective nature may lead to inevitable selection bias even after PSM. Besides, the SEER database has the inherent limitation to provide all clinically significant variables for CDS analysis, including performance status, preoperative comorbidities, postoperative complications and subsequent treatments. It is difficult to parse out patients’ decision-making processes in “real-world” clinical practice. In addition, SEER cannot provide other related factors such as characteristics of the surgeons that probably influence receipt or refusal of CDS. Therefore, we should admit the difficulty to uncover the real truth of the past and accurately specify "recommended" and "accepted" CDS treatment. Moreover, our observational study provided insufficient information to clearly explain the causal relationship between sociodemographic factors and CDS refusal in PCa patients. Additionally, our study did not involve the medical insurance status and investigate how patients’ income and their ability to afford surgical fees, partially reduced the confidence power. On top of that, we conducted two adjusted models to explore the value of CDSA on CSM and OM. Unfortunately, due to the missing data in SEER database, we could not enroll several potentially related factors for analysis, such as PSA, Gleason score and TNM stage. Detailly, disease-related factors such as GS, PSA, and T stage was not adjusted with PSM in this study, which is a limitation to permit valid comparisons. Despite this, the main goal of this study is to uncover sociodemographic factors and their impacts on survival associated with cancer-directed surgeries refusal. The impacts of PSA, GS, and AJCC stages on survival time have been well discussed in studies^27–29^. Additionally, we did not perform subset analyses to identify whether CDS impacts on survival outcomes in GS 6 or ≤ T2 disease. GS 6 or ≤ T2 stage represents localised prostate cancer and CDS is one of the most effective treatments for this type of prostate cancer according to EAU guideline and other guidelines. These patients with CDS presented a longer survival time as supported by numerous studies^30,31^. In spite of these limitations, however, our present study was one of the largest SEER-based analysis to identify predictors for patients’ CDS refusal and subsequent effect on cancer survival. One strength of this study was the application of a series of statistical analyses such as PSM to mitigate limitations. Notably, our study shined a spotlight on physicians’ key role in patients’ decision-making process, providing valuable information for patient-doctor relationships and communication.

## Conclusions

In conclusion, our study revealed that physicians may weigh a host of socio demographic and clinical factors prior to making a CDS recommendation to PCa patients. Patients’ acceptance of recommended CDS was potentially modifiable by certain sociodemographic factors. Physicians, especially urologists, should fully consider the hindrances behind patient’s refusal of recommended CDS, thus improving patient-doctor shared decision-making, guiding patients toward the best alternative and achieving better outcomes. Further studies are necessitated to confirm the generality of our results.

## Data Availability

The datasets generated for this study are available on request to the corresponding author.
